# Toxicological and biochemical responses of the earthworm Eisenia fetida to cyanobacteria toxins

**DOI:** 10.1038/s41598-017-16267-8

**Published:** 2017-11-21

**Authors:** Qing Cao, Alan D. Steinman, Lei Yao, Liqiang Xie

**Affiliations:** 1 State Key Laboratory of Lake Science and Environment, Nanjing Institute of Geography and Limnology, Chinese Academy of Sciences, 73 East Beijing Road, Nanjing, 210008 China; 2 University of Chinese Academy of Sciences, Beijing, 100049 China; 3 Annis Water Resources Institute, Grand Valley State University, 740 West Shoreline Drive, Muskegon, MI 49441 USA

## Abstract

Irrigation with eutrophic water containing cyanobacteria toxins poses a potential risk to soil animals. To evaluate ecotoxicological effect of microcystins (MCs) on earthworms, filter paper acute toxicity test, avoidance test and a 14-d artificial soil test were carried out. No acute toxicity was found in the filter paper test, and earthworms showed no avoidance response to MCs exposure. In the artificial soil test, *Eisenia fetida* were allowed to grow in presence or absence of MCs (0, 1, 10, 100, 1000 μg kg^−1^ of soil) for 1, 7, and 14 d. Results showed that MCs could bioaccumulated in earthworm. A stimulatory effect on catalase and glutathione oxidase activities induced by MCs was found on day 1, and both of them were significantly inhibited at 100 and 1000 μg kg^−1^ on days 14. The superoxide dismutase activity was relatively insensitive. Significant increase of malondialdehyde content and decrease of neutral red retention time were observed at 100 and 1000 μg kg^−1^ on days 7 and 14. Our results suggest that MCs induces oxidative stress on earthworms, which leads to disruption of the antioxidant system and lipid peroxidation, as well as alterations in lysosomal membrane stability.

## Introduction

In eutrophic fresh water lakes, a group of toxic compounds are produced by some bloom-forming cyanobacteria. The most prominent of these cyanotoxins are microcystins (MCs). Owing to their potential carcinogenicity, MCs can negatively affect both public health and fundamental ecological processes^1^. MCs exposure induced thyroid endocrine disruption and transgenerational effects of developmental neurotoxicity in zebrafish offspring as well as in embryo^2–4^. More than 200 different structural analogues of MCs, with a range of molecular weights from 882 to 1116 Da, have been identified from cyanobacterial blooms and cultures^5,6^. Microcystin-leucine-arginine (MC-LR) is found to be the most common and potent analogue, followed by Microcystin-arginine-arginine (MC-RR) and Microcystin-tyrosine-arginine (MC-YR)^7^. With the increasing prevalence of these cyanobacteria blooms, more attention is being paid to impacts caused by MCs.

MCs can be brought into contact with soils by irrigation source water that contains cyanobacteria; in addition, cyanobacterial blooms can be applied directly to soil as organic fertilizer after being intentionally harvested from lakes^8–11^. Total MC concentrations in surface waters varied from less than 1 μg L^−1^ to 29, 000 μg L^−1^ ^12–16^. However, high concentrations of MC would be from very dense cyanobacterial biomass, and the concentration of MCs in most of the water samples were less than 100 μg L^−1^ ^17–19^. Although the MCs concentration in field samples collected near Dianchi Lake were relatively low, ranged from 1.43 to 21.3 μg kg^−1^ ^20^, high concentrations of MC will occur by irrigation with water containing dense cyanobacterial biomass. Moreover, once come in contact with soil, MCs can persist in soils with a half-life ranging from several days to several months^21,22^, depending on the efficiency of degradation, mainly photolysis and bacterial degradation. Studies on the absorption of MCs in soils are rare. It is suggested that organic carbon content and clay content in soils were important for the adsorption of MCs, and sandy soil was incapable of the removal of MCs^23,24^. However, Eynard *et al*.^25^ indicated that soil was unable to protect groundwater from surface water that contains cyanotoxins. Therefore, there is a possibility that soils adjacent to lakes and reservoirs may be contaminated by MCs. And the low absorption of MCs in soils could lead to their high bioavailability to soil organisms, animals and plants. However, very few studies have examined the impact of MCs on soils, especially on soil animals, which play important roles in the soil ecosystem. A recent study reported the toxic effects of MC-LR on earthworms^26^. In their study, MC-LR caused reproductive, biochemical, and cellular toxicity to earthworms. Li *et al*.^27^ used the nematode *Caenorhabditis elegans* as a model animal for assessing the toxicity induced by microcystin-LR. Reduced life span, extended generation time, decreased brood size, delayed development and suppressed locomotion behavior were found in *C. elegans* after exposed to MC-LR. *Caenorhabditis elegans* were also used for a chemotactic behavior study induced by MCs^28^. They found that MCs altered AWA but not AWC sensory neuron function via mechanisms other than protein phosphatases inhibition. However, the survival or reproduction of springtail *Folsomia candida* was not affected by water bloom biomass at concentration up to 4 g kg^−1^ DW soil^29^. As it is known to us, earthworms play vital roles in soil system, such as nutrient cycling, climate, water purification, remediation and restoration^30^. Moreover, earthworms can be used as bioindicators of various soil contaminations due to their sensitivity to pollutants^31,32^. Antioxidant enzymes in earthworms, which protect cells against adverse effects of reactive oxygen species (ROS), are used as indicators for evaluating the environmental effect of soil pollutants^33–35^. As a sensitive cellular biomarker, lysosomal membrane stability can provide valuable information on cellular damage^36^. Among earthworms, *Eisenia fetida* is sensitive to various toxicants and can be cultured easily under laboratory conditions. So it has been used as a standard animal for toxicology experiment^37^. In the present study, *E. fetida* was used for assessing the toxicological effects of MCs. In addition, biochemical responses in earthworms can be used as early warning indicators of soil contamination.

Cyanobacterial blooms have become more frequent in recent years in many large freshwater lakes and reservoirs. Surface water containing MCs used as irrigation source were reported all over the world, including but not limited to China^16^, India^38^, Australia^39^, United States^40^ and Finland^12^. Consequently, it is possible that large areas of farmland near contaminated lakes are exposed to irrigation source water containing cyanobacteria blooms. In the present study, we measured mortality, avoidance responses, bioaccumulation, biochemical responses, and lysosomal membrane stability in earthworms exposed to different concentrations of cyanobacteria crude extract. These findings help to give a better understanding of the potential environmental risk of irrigation with cyanobacteria bloom into agricultural soils.

## Results

### Filter paper assay

There was no significant effect of microcystins on earthworms with filter paper test. Survival was 100% in all treatments after the 72 h exposure. In addition, earthworm weight was not significantly affected by MCs concentration (ANOVA, p > 0.05; data not shown), nor were morphological abnormalities observed in any of the treatments.

### Avoidance responses

Earthworm net avoidance response was not significantly affected by MCs treatment in soils. (ANOVA, p > 0.05; data not shown).

### Bioaccumulation

There was no evidence of MCs bioaccumulation in earthworms from control and 1 μg kg^−1^ treatments. For 10, 100 and 1000 μg kg^−1^ treatments, MCs were not detected on day 1, but increased along with increasing MCs dose on days 7 and 14 (Table 1). A sharp increase was observed from day 1 to day 14 for 100 and 1000 μg kg^−1^ treatments. MCs level in 10 μg kg^−1^ treatment increased relatively slow. At days 14, MC concentrations in earthworms were 0.18, 0.40 and 0.63 μg g^−1^ in 10, 100 and 1000 μg kg^−1^ treatments, respectively. MCs in artificial soils were also determined. After 14 d exposure, MCs content in soils decreased by 16%, 11%, 17% and 19% at 1, 10, 100 and 1000 μg kg^−1^ treatments, respectively (data not shown).

**Table 1:** MC concentrations in earthworm body after 1, 7 and 14 d exposure.

	10 μg kg^−1^	100 μg kg^−1^	1000 μg kg^−1^
1 d	ND	ND	ND
7 d	0.13 ± 0.02^**^	0.27 ± 0.06^**^	0.34 ± 0.04^**^
14 d	0.18 ± 0.01^**^	0.40 ± 0.09^**^	0.63 ± 0.04^**^

Data were showed as mean values (n = 3) ± SD. The unit for MCs concentration in earthworms are μg g^−1^. MCs in control and 1 μg kg^−1^ treatment were not detected. Significantly different from the control: *P < 0.05, **P < 0.01.

### Biochemical responses

**Figure 1:**
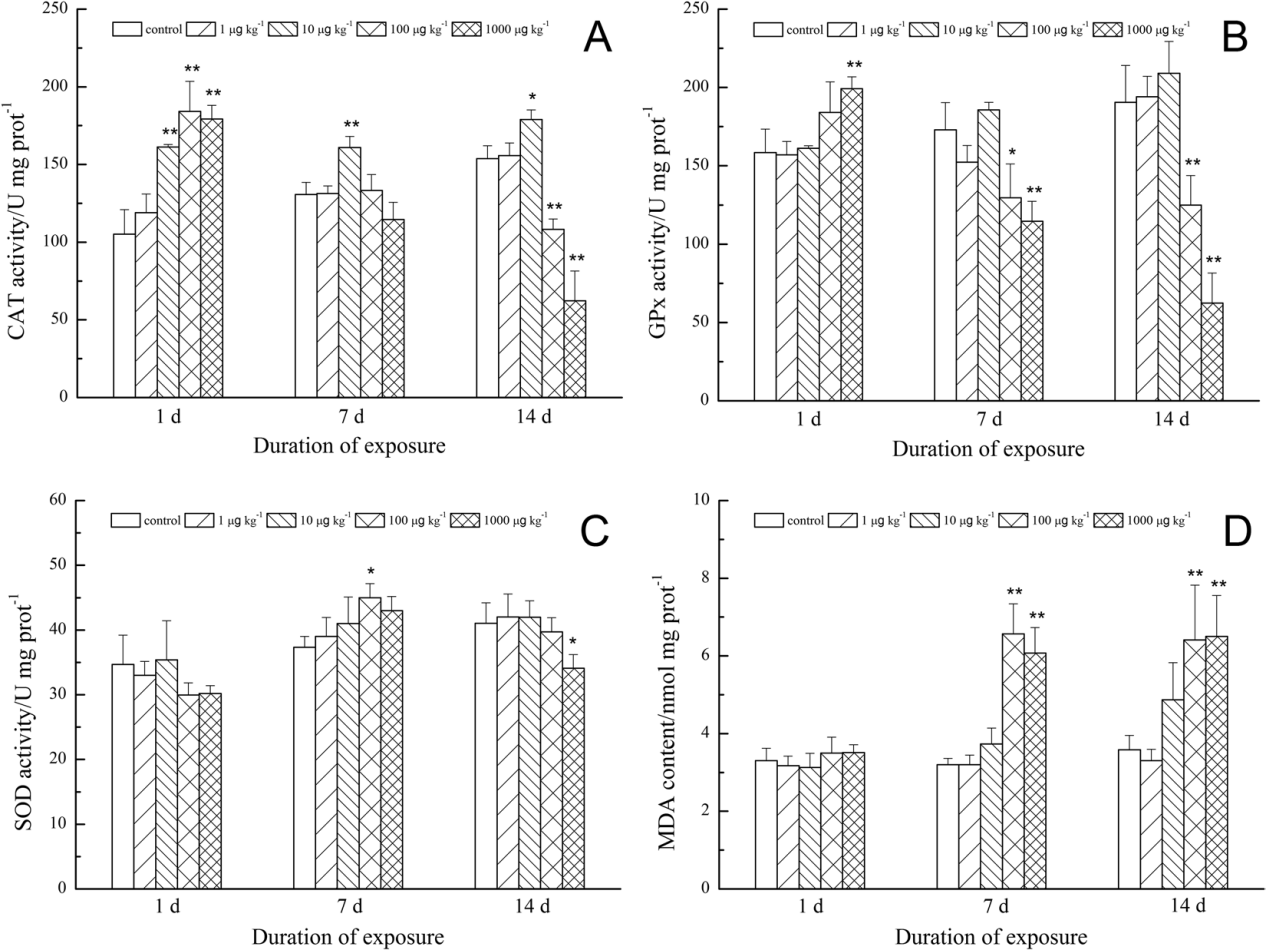
Effect of MCs on CAT activity (**A**), GPx activity (**B**), SOD activity (**C**) and MDA content (**D**). Data were showed as mean values (n = 3) ± SD. Significantly different from the control: *P < 0.05, **P < 0.01.

The treatment with 1 µg MCs kg^−1^ did not affect CAT activity regardless of exposure time. However, CAT activity responded quickly to the application of higher concentrations of MC; CAT activity at 10, 100, and 1,000 μg kg^−1^ increased significantly after 1 d (Fig. 1A). However, after a 7 d exposure, the only CAT activity that was significantly different from the controls was the 10 μg kg^−1^ treatment, similar to day 1 response. After 14 days of exposure, CAT activity at 10 μg kg^−1^ was still statistically greater than the controls, but significantly lower values of CAT activity were found at 100 and 1,000 μg kg^−1^.

The effect of MCs on GPx activity was concentration and time-dependent (Fig. 1B). Both 1 and 10 μg kg^−1^ treatments had no effect on GPx activity compared to controls, regardless of exposure time. However, GPx activity in the 1,000 μg kg^−1^ treatment was significantly higher than the controls after 1 d exposure, but then was significantly lower at days 7 and 14. GPx activity at 100 μg kg^−1^ was not significantly different from the control at day 1, but similar to the 1,000 μg kg^−1^ treatment, and was significantly lower than the controls at days 7 and 14.

SOD activity was relatively insensitive to MCs application (Fig. 1C). SOD activity at 100 μg kg^−1^ was enhanced after 7 d, while it was significantly inhibited at 1,000 μg kg^−1^ after 14 d compared to the controls. No significant differences were found in the other treatments.

The MDA content of earthworms was not significantly affected after a 1 d exposNure, while significant increases were measured in 100 μg kg^−1^ and 1,000 μg kg^−1^ treatments after 7 and 14 d of exposure (Fig. 1D). The MDA content at the two highest MC concentrations on days 7 and 14 was almost two times higher than the controls.

Significant influence of dose and duration of exposure on all studied biochemical responses was revealed by multivariate analysis. The CAT and GPx activities and MDA content were significantly affected by both dose and duration of exposure, while SOD activity wasonly affected by duration (Table 2).

**Table 2:** Results of ANOVA tests on the biochemical responses of earthworms exposed to MCs.

	Dose			Duration			Dose × Duration		
	df	F	P	df	F	P	df	F	P
CAT activity	4	16.296	<0.0001^**^	2	7.938	0.0017^*^	8	22.954	<0.0001^**^
GPx activity	4	14.025	<0.0001^**^	2	4.905	0.0144^*^	8	13.551	<0.0001^**^
SOD activity	4	1.120	0.3655	2	21.674	<0.0001^**^	8	2.239	0.0525
MDA content	4	17.682	<0.0001^**^	2	17.645	<0.0001^**^	8	3.573	0.0051^**^

Dose or the duration of exposure had a significant effect (*p < 0.05, **p < 0.01). df, degree of freedom.

### Lysosomal membrane stability

**Figure 2:**
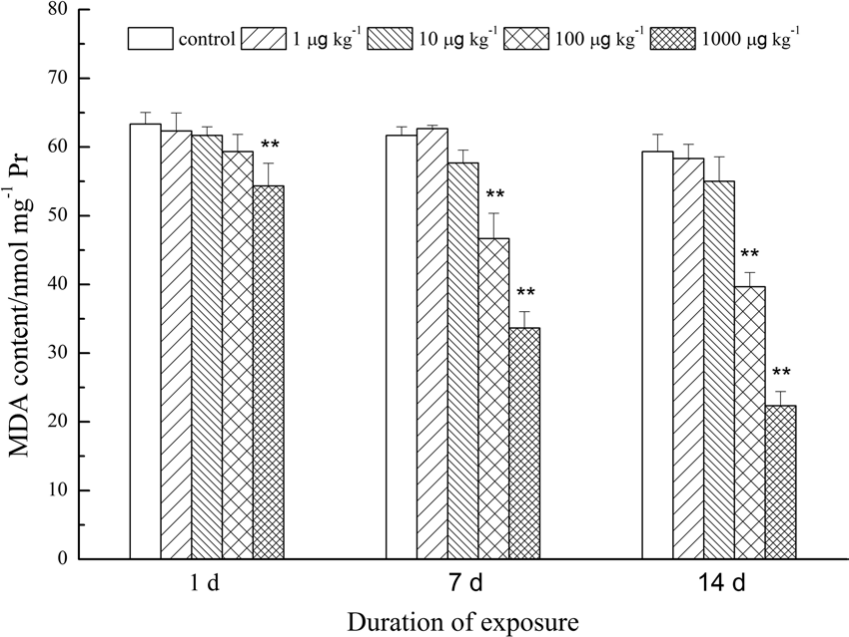
Effect of MCs on lysosomal membrane stability in coelomocytes of earthworms, as evaluated by the NR retention time assay. Data were showed as mean values (n = 3) ± SD. Significantly different from the control: *P < 0.05, **P < 0.01.

MCs had a negative effect on the NRRT of earthworms at the highest concentration on day 1, but no other concentration had a significant effect compared to controls on that day (Fig. 2). A significant reduction in NRRT was observed with both 100 and 1,000 μg kg^−1^ treatments on days 7 and 14, with reductions of 45% and 62%, respectively.

## Discussion

In this study, we found no evidence that MCs are acutely toxic to *Eisenia fetida*. There was no mortality or morphological changes even at an exposure of 10,000 μg L^−1^ MCs. Furthermore, no avoidance response was found in any of the MC-treated soils. In contrast to our findings, Wen *et al*.^26^ reported that the LC_50_ of MC-LR was 0.149 μg cm^−1^ at 72 h based on a filter paper test on *Eisenia fetida*. The explanation for such different might be due to the earthworms (300–400 mg) used in their study is smaller than ours (~500 mg), and MCs are more toxic to younger earthworms. Toxic effects of MCs on aquatic animals have also been reported before. Oberemm *et al*.^41^ found a dose-dependent increase of mortality rate of chub (*Leuciscus cephalus*) after exposure to 0.5, 5, or 50 μg MC-RR or MC-LR L^−1^. Liu *et al*.^42^ also reported mortality (LC_50_ = 593.3 μg/L) of juvenile loach (*Misguruns mizolepis*) after exposure to 1, 3, 10, 100, or 1,000 μg MC-LR L^−1^ after 7 d. Various abnormalities, as well as dose- and time-dependent survival rate was found after micro injection of 300, 750, and 900 nM MC-LR into zebrafish (*Danio rerio*)^43^. However, some animals such as mollusks showed tolerance to cyanotoxins. Vasconcelos^44^ reported that less than 1% mortality of mussel (*Mytilus galloprovincialis*) was found after fed on a toxic *M. aeruginosa* strain for 16 days. During this period mussels accumulated up to 10.5 µg g^−1^ MCs dry mussel weight. *Anodonta cygnea* was also found to be able to accumulate high levels of microcystins produced by *Oscillatoria agardii HII* without any visible damage^45^. There are no specific carriers that carry MCs, which are relatively lipophobic, into hepatopancreatic cells^46^. Moreover, MCs used in the present was cyanobaterial extract contained mainly MC-RR, which is less toxic than the most common congener MC-LR in a mice test^47^. Thus, toxicity of different MC congeners in earthworms should be explored in the future. Besides, in view of the toxicity of MCs to aquatic animals, short- and long-term effects of MCs on other soil organisms should also be evaluated. Our results showed that concentrations of MCs in earthworms were high when MC concentrations in treatments were above 10 μg kg^−1^, suggesting that MCs may bioaccumulate in earthworms. With less microbes and no light in the artificial condition, which are main factors for MCs degradation^1^, MCs degradation in artificial soil is much lower than that in natural soil. Degradation of MCs in the artificial soil is less than 20% at the end of the exposure experiment. So the bioaccumulation of MCs in earthworms in natural soil should be re-evaluated due to different soil type and different quantity of MCs. Moreover, we were unable to completely remove earthworm gut contents, even when gut contents of earthworms were voided with wet filter paper for 24 h after exposure. Hence, we suggest that the biological responses (such as response of the antioxidant system) observed in our study are better indicators of impacts from MCs than tissue levels.

The biochemical indicators varied with doses of MCs and duration of exposure. One of the toxicity mechanisms of MCs to animals is oxidative stress^48^. When cellular antioxidant defense fail to remove excessive ROS, oxidative stress results in organisms^49^. CAT, which eliminates hydrogen-peroxide, plays an important role in anti-oxidant systems. In our study, CAT activity in *E. fetida* was sensitive to MCs exposure. A significant increase was found at 10, 100, 1,000 μg kg^−1^ after only 1 d exposure, indicating that MCs lead to oxidative stress in this earthworm species^50^. However, the stimulatory effect of 100 and 1,000 μg kg^−1^ in our study disappeared after 7 d, with a significant decrease observed after 14 d exposure. The same time-dependent effect was found in crabs, Pinho *et al*.^51^ showed that CAT activity increased after exposed to 5.32 mg MCs kg^−1^ day^−1^ for 2 days, whereas decreased at day 7. A possible explanation is that the production of H_2_O_2_ at the early stage may induce an increase of CAT activity_,_ whereas the natural antioxidant defenses may have become saturated as incubation time lengthened, resulting in accumulation of ROS in earthworms, thereby inhibiting CAT activity^52,53^. As antioxidant system vary with animal species, different result was found in freshwater clam *Diplodon chilensis patagonicus*, in which a continuous stimulatory effect of CAT was observed after exposed to an estimated mean weekly dose of 0.625 mg MCLR g^−1^ clam for six weeks^54^. Leão *et al*.^55^ found decreased CAT activity in an estuarine worm (*Laeonereis acuta*) after 48 h exposure to 2 mg mL^−1^ lyophilized cells of toxic *M. aeruginosa*. Moreover, we detected all the biochemical indicators with whole earthworm tissue with no distinction of different organs. Lower CAT level was found in gills than other organs (liver and kidney) of tilapia^56^. So this may raise questions on the difference of antioxidant response in different organs. Therefore, alternation of antioxidant enzyme activities in different earthworm organs after exposed to MCs should be studied in the future.

GPx is another enzyme that can remove H_2_O_2_ in organisms by using reduced glutathione as a hydrogen donor. Similar to CAT activity, a stimulatory effect was observed at 1,000 μg kg^−1^ after 1 d exposure, and a significant inhibition at 100 and 1,000 μg kg^−1^ began after 7 d exposure, which continued through day 14. Stimulatory effect of GPx activity was also observed in embryos of zebrafish exposed to 0.5 μg L^−1^ MC-LR^57^, and in hepatocytes of *C. carpio* exposed to 10 μg L^−1^ MC-LR^58^. However, GPx activity was increased in liver and intestine of *Corydoras paleatus* exposed to 2 μg L^−1^ MC-RR, whereas strongly inhibited in gills at all tested concentrations^59^. Same differential impact vary with organs was found in tilapia^56^. Similar to CAT, as previously discussed, antioxidant response in different earthworm organs should be re-evaluated. Interestingly, same as our results, a previous study on earthworms^60^ also found that GPx activity was induced by naphthenic acids after 1 d but inhibited after 14 d of exposure. The reasons might be that (i) GPx activity was inhibited by excessive ROS in cells; and (ii) large amounts of glutathione (GSH) were consumed to remove ROS.

SOD also plays a vital part in eliminating ROS. The ^·^superoxide anion radical (O_2_^−^) in the cell is transformed to H_2_O_2_ by SOD and then H_2_O_2_ is transformed to harmless H_2_O and O_2_ by CAT and GPx under oxidative stress^61,62^. Unlike CAT and GPx activity, no significant change was found after 1 d in our study. A stimulatory effect was found only at 100 μg kg^−1^ after 7 d, which probably was caused by O_2_^−^ accumulation^51^. Inhibition was found at 1,000 μg kg^−1^ after 14 d. The possible reason is that hydrogen peroxide, peroxyl radicals and singlet oxygen induce inactivation of SOD or the highly reactive superoxide was eliminated^63–66^. Increase as well as decrease of SOD activity in organs after treated with MCs were found by previous studies^56,67–69^. Similar to CAT and GPx activities, SOD activity varies with animal species.

MDA results when unsaturated fatty acids react with free radicals in cellular membranes. It is used frequently as a sensitive indicator that reflects lipid peroxidation and indicates intracellular injury^70^. Various contaminants are known to induce lipid peroxidation due to excessive ROS^71^. In our study, MDA content in earthworms was significantly higher than that observed in controls after 7 d and 14 d of exposure to higher concentrations of MC (100 and 1,000 μg kg^−1^). These results suggest that MCs lead to lipid peroxidation and induce oxidative injury in earthworms.

Oxidative stress in animals and plants caused by MCs has been reported in recent years. Generation of ROS and disruption of mitochondrial electron transport chain were found in MC-treated rats^48^. Oxidative stress caused by excessive ROS production was found in several MC-treated aquatic and terrestrial organisms, and an increase in lipid peroxidation was found as well^51^. Regarding plants, SOD activity in rape (*Brassica napus* L.) decreased as toxin concentration increased, while activity was stimulated in rice (*Oryza sativa* L.)^72^. Stimulatory effects of MCs on SOD and GPx activities also were found by Chen *et al*.^10^ and Gehringer *et al*.^73^ in apple trees (*Malus pumila)* and garden cress (*Lepidium sativum)*, respectively. Oxidative stress was found in several MC-treated animals and plants, changes in antioxidative activity varying with time and tested species.

Our results demonstrated that MCs can induce oxidative stress in earthworms, as observed in other animals and plants, which can lead to the disruption of the antioxidant system and lipid peroxidation. Nevertheless, the duration of our study was only 14 d; we recommend that earthworm responses to MCs be evaluated over longer time periods and in natural conditions. Generally, impaired lysosomal reactions precede cell and tissue pathology; therefore, lysosomal systems can respond quickly to pollutant exposure^74^. LMS is an early indicator of various environmental contaminants, and LMS in earthworm coelomocytes is often evaluated by the NR retention assay^32,36,75^. In the present study, high concentrations of MC significantly decreased LMS in earthworm coelomocytes. A same inhibition effect of NRRT was reported by Wen *et al*.^26^ in MC-LR treated earthworms. However, the mechanism causing alternations in LMS is still unknown. Evidence suggests that ROS produced in the cellular system affects various cellular organelles^76,77^. Consequently, generation of ROS induced by MCs may be the cause of alterations in LMS. This putative mechanism should be explored in future studies to better understand the impact of MCs on LMS on earthworm coelomocytes.

Soils irrigated with cyanobacteria-containing water may suffer varying degrees of MCs contamination, which could pose a threat to earthworms in the soil. Although no significant effect on earthworms was observed at low MC concentrations treatment, effects on earthworms after long-time and/or repeatedly contact with MCs are still unknown. In the artificial soil test, earthworms are cultivated in artificial soil according to OECD^78^. Although the composition of artificial soil was set to mimic natural soil, the microbial composition of artificial soil is much different from that in natural soil. Moreover microorganisms play important roles in degrading MCs in soils. The implication for natural soil systems is unclear, so field experiments are needed in the future.

## Materials and Methods

### Earthworms

Earthworms were purchased from a local earthworm farm in Nanjing. Adult earthworms weighing about 500 mg with well-developed clitella were selected. To void gut contents, earthworms were placed on damp filter paper in Petri dishes for 24 h before testing.

### Extraction and analysis of microcystin variants

In the present study, we used a natural cyanobacterial bloom extract so as to mimic natural toxic conditions with multiple MCs. In order to prevent interference from impurities, the extract was purified with an Oasis HLB extraction cartridge (Waters). The method of extraction and purification was modified from Harada *et al*.^79^. Cyanobacterial cells obtained from Dianchi Lake (~1 g DW) were homogenized with 25 mL of 5% (v/v) aqueous acetic acid after freeze-drying. The homogenate was subjected to an ultrasonic bath for 5 min, and then centrifuged at 10,000 r min^−1^ at 4 °C for 15 min. After re-extracting the residue two more times as before, all the supernatant was collected and then applied to 5 g HLB extraction cartridge. The cartridge with toxin was rinsed with 50 mL of 5% (v/v) aqueous methanol. Subsequently, the cartridge was eluted with 100 mL 100% aqueous methanol. The eluant was evaporated to dryness, and then 10 mL deionized water was used to dissolve the toxin. Combined toxin-containing solutions were stored at −40 °C before use. According to Corbel *et al*.^80^, microcystin variants were analyzed by ultra-high-performance liquid chromatography tandem mass spectrometry (UHPLC/MS), using a Waters Acquity UPLC system coupled to a triple-quadruple mass spectrometer (TQD, Waters, France) via an electrospray ionization (ESI) interface. MC-LR (14.92 mg L^−1^), MC-YR (5.90 mg L^−1^) and MC-RR (61.61 mg L^−1^) were found in the extract after the analysis. The toxin extract was diluted as needed for subsequent use.

### Filter paper acute toxicity test

The acute toxicity test was conducted according to OECD guidelines^78,81^. Considering there are no previous studies reporting the toxicity of MCs on earthworms, a wide range of MC concentrations was used for the acute toxicity test (MCs concentration, 0.1, 1, 10, 100, 1,000, 10,000 μg L^−1^). After placing single pieces of filter paper on the bottom of Petri dishes, 1 mL of each MC treatment concentration or deionised water (controls) was added to wet the entire filter paper. Then one earthworm was randomly placed in each Petri dish. Five replicates were used for each treatment. The Petri dishes were incubated in the dark at 20 ± 1 °C for 72 h and the mortality was recorded every 24 h; earthworm weight and morphological changes also were observed.

### Avoidance test

According to ISO guideline^82^, avoidance tests were performed in plastic containers with three replicates for each treatment. Soils used in the test was collected (0–15 cm) from an area adjacent to Lake Taihu at Suzhou, with no known previous history of MCs application. Some of the physicochemical properties are as follows: 27.9% sand, 31.0% silt, 41.2% clay, pH 7.46, 9.91 g organic matter kg^−1^ soil, 0.78 g total nitrogen kg^−1^ soil, 15.4 mg available P kg^−1^ soil, 150 mg available K kg^−1^ soil. After taken back to the laboratory, the soil samples were homogenized and passed through a 2-mm sieve to sift out roots and other large debris. The soil samples were used for physico-chemical analysis after air-dried at 25 °C in 48 h. The remainders were kept fresh at 4 °C for avoidance test. Contaminated soil was produced by thoroughly mixing soil and MCs containing deionized water with final treatment concentrations of 1, 10, 100, 1,000 μg kg^−1^ dry soil. Control soil received an equal amount of deionized water. All treatments were adjusted to 60% of maximum water-holding capacity. Briefly, the container was filled half with contaminated soil and half with control soil at each end. After placing ten earthworms on the soil surface along the transition line between control and contaminated soil in each test container, perforated plastic films were used to cover the containers. The containers were incubated in the dark at 20 ± 1 °C for 48 h. The numbers of earthworms in the control vs. contaminated sections of each container were recorded after incubation. The net avoidance response (NR) was determined with the following formula: NR = (C − T)/N × 100%, where C, T, and N represent the numbers of earthworms in the control soil (C), the contaminated treatment soil (T), and the total number of earthworms (N), respectively.

### Artificial soil test

A 14-day toxicity test was performed in artificial soil according to OECD^78^ guidelines. The artificial soil was composed of 10% finely ground sphagnum peat, 20% kaolin clay and 70% industrial sand with 35% water content and 7.0 ± 0.2 pH value. Before the test, earthworms were acclimated for one week in untreated artificial soil at a controlled temperature of 20 ± 1 °C with 80–85% relative humidity under 400–800 lux of continuous light. Different amount of MCs were added in the soil to make a final MC concentrations of 1, 10, 100, 1,000 μg kg^−1^ dry soil. The control soil received an equivalent amount of distilled water. The MCs concentrations in the artificial soil prior to the exposure were 0.65, 8.47, 90.1 and 958.3 μg kg^−1^ dry soil. Twelve earthworms were added to each 1 L wide-mouth bottle filled with 500 g contaminated soil. Each soil treatment included three replicates. The bottles were sealed with porous plastic film, allowing exchange of air, and incubated at 20 ± 1 °C with 80–85% relative humidity under 400–800 lux of continuous light for 14 days. Four earthworms were randomly collected on days 1, 7, and 14 following application of MCs. Prior to analysis, the gut contents of all the earthworms were voided as mentioned above. No mortality was observed during the experiment.

### Analysis of MCs in earthworms

Earthworms were lyophilized after voiding gut contents. MCs in lyophilized earthworms were determined as described by Xie and Park^83^. The limit of detection (LOD) for MC-LR, MC-RR and MC-YR are 0.022, 0.024 and 0.030 μg kg^−1^, respectively. The recovery rate ranged from 83.5% to 97.5% (RSD ranged from 0.1%–2.3%). Briefly, lyophilized earthworms were homogenized with 10 mL of BuOH:MeOH:H_2_O (1:4:15, in volume) after ground with a mortar. The homogenate was subjected to an ultrasonic bath for 3 min, and then centrifuged at 4,000 r min^−1^ at room temperature for 20 min. After re-extracting the residue two more times as before, all the supernatant was collected and then applied to 500 mg Oasis HLB extraction cartridge. The cartridge with toxin was rinsed with 20 mL of 5% (v/v) aqueous methanol. Subsequently, the cartridge was eluted with 15 mL 100% aqueous methanol. The eluant was evaporated to dryness, and then 2 mL 100% aqueous methanol was used to dissolve the toxin. The dissolved toxin was then applied to a silica gel cartridge (2 g)/plus silica gel (0.69 g) tandem cartridge. The cartridge with toxin was rinsed with 20 mL of 100% aqueous methanol, and eluted with 20 mL 70% aqueous methanol. The eluant was evaporated to dryness and the residue was dissolved in 0.1 mL 100% aqueous methanol. The toxin solutions obtained were stored at −40 °C for UHPLC/MS detection.

### Analysis of MCs in artificial soils

The artificial soil was lyophilized after collected at the end of the test. MCs in soils were determined as described by Li *et al*.^20^ with slight modification. The LOD for MC-LR, MC-RR and MC-YR are 0.25, 0.25 and 0.50 μg kg^−1^, respectively. The recovery rate ranged from 54.9% to 97.4% (RSD ranged from 4.3%^−1^6.9%). Briefly, lyophilized soil samples were extracted 3 times with 30 mL of 0.1 M EDTA-0.1 M Na_4_P_2_O_7_ with 10 min ultrasonic bath after ground with a mortar. The homogenate was then centrifuged at 4,000 g for 10 min. After turning the pH value of the supernatant to pH 3 with TFA, the solution was centrifuged again with the same condition. Then the aqueous extractions were applied to an Oasis HLB extraction cartridge. The cartridge with toxin was rinsed with 15 mL of 20% (v/v) aqueous methanol, and eluted with 10 mL 90% aqueous methanol. The eluant was evaporated to dryness and the residue was dissolved in 0.1 mL 100% aqueous methanol. The toxin solutions obtained were stored at −40 °C for UHPLC/MS detection.

### Biochemical assays

Enzymes were extracted according to Mishra and Dash^84^. Earthworms were ground under ice-cold conditions in 0.85% NaCl solution (1:9, w/v) with a prechilled mortar. The homogenate was centrifuged at 3,000 rpm at 4 °C for 10 min. The supernatants were used for various analyses. Several test kits (Nanjing Jiancheng, China) including Catalase (CAT) assay kit (Ammonium molybdate method), Glutathione Peroxidase (GPx) assay kit (Colorimetric method), Total Superoxide Dismutase (T-SOD) assay kit (Hydroxylamine method), Total protein quantitative assay kit and Malondialdehyde (MDA) assay kit (TBA method) were used to measure CAT activity, GPx activity, total SOD activity, protein content and MDA content, respectively.

### Neutral red retention assay

The non-invasive extrusion method was used to obtain earthworm coelomocytes^85^. The extrusion medium (10 mg ml^−1^ guaiacol glyceryl ether, 0.85% saline, 5% ethanol, 2.5 mg ml^−1^ EDTA,) was used to rinse earthworms. Coelomocytes secreted in the medium were washed three times with 100 mM PBS (pH 7.3) to remove mucous. Neutral red (NR) retention assay was used to evaluate lysosomal membrane stability of coelomocytes as described by Lowe and Pipe^86^.

### Statistical analysis

SPSS software (SPSS 22.0, Inc., 2013) was used for statistical analyses. Data were expressed as mean ± SD. One-way ANOVA followed by the Duncan test were used to test for differences between different exposure levels and the control. Means of biochemical indicators were compared by two-way ANOVA with dose and duration treatments as independent variables. The criteria for significance were set at p < 0.01 and p < 0.05.

### Data availability

All data generated or analysed during this study are included in this published article (and its Supplementary Information files).

## Conclusion

The results obtained from various tests and multiple biomarkers lead us to conclude that: (1) MCs do not induce acute toxicity in earthworms; (2) earthworms showed no avoidance response to MCs exposure; (3) MCs were bioaccumulated in earthworms after exposed to high concentrations of MCs; and (4) MCs induced oxidative stress in earthworms, which lead to disruption of the antioxidant system and lipid peroxidation, as well as alterations in LMS. More studies are encouraged to figure out the thorough impact of MCs on soil animals.
